# Use of analgesics during pregnancy: patterns of use and knowledge of medication safety among women in Serbia

**DOI:** 10.3389/fphar.2026.1874369

**Published:** 2026-06-18

**Authors:** Nemanja Maletin, Milica Kusturin, Nikola Denda, Aleksandar Rašković, Milica Paut Kusturica

**Affiliations:** 1 Department of Pharmacology, Toxicology and Clinical Pharmacology, Faculty of Medicine Novi Sad, University of Novi Sad, Novi Sad, Serbia; 2 Clinic of Internal Oncology, Oncology Institute of Vojvodina, Sremska Kamenica, Serbia; 3 Clinic for Eye Diseases, University Clinical Center of Vojvodina, Novi Sad, Serbia

**Keywords:** analgesics, knowledge, medication safety, pregnancy, self-medication

## Abstract

**Background:**

Analgesics are commonly used during pregnancy, and their safe use depends on the type of drug, timing of exposure, and appropriateness of use. This study assessed patterns of analgesic use before and during pregnancy and evaluated women’s knowledge and perceptions regarding analgesic safety during pregnancy in Serbia.

**Methods:**

A cross-sectional study was conducted using an electronically distributed, self-administered questionnaire adapted from a previously published instrument. The survey included socio-demographic and reproductive characteristics, patterns of analgesic use, and a 19-item knowledge inventory. Knowledge scores were categorized as low (<10 points) or high (≥10 points). Descriptive statistics and logistic regression were used for analysis.

**Results:**

A total of 538 women participated. Overall, 51.7% reported analgesic use during pregnancy, and among these, 50.7% reported use during the first trimester. Paracetamol was the most commonly used analgesic during pregnancy (93.9%). Analgesic use without prior consultation was reported by 32.0% of women who used analgesics during pregnancy. Before pregnancy, 83.1% reported analgesic use. Although 69.3% reported having received information about adverse effects, important uncertainty remained regarding NSAID classification and safety. Overall, 65.1% of respondents had a low level of knowledge. Health-professional background was strongly associated with a high level of knowledge (adjusted OR 12.468, 95% CI 7.502–20.724; p < 0.001).

**Conclusion:**

Analgesic use during pregnancy was common, including use without prior consultation. Despite recognizing paracetamol as the preferred analgesic, many women had limited knowledge regarding analgesic safety, particularly NSAIDs, in pregnancy. These findings support the need for improved counselling and targeted education on medication safety during pregnancy.

## 1 Introduction

Analgesics are among the most commonly used medications worldwide and are frequently used by women of reproductive age, including during pregnancy. Because many analgesics are available without prescription, their use may be influenced not only by medical advice, but also by self-medication, previous personal experience, and informal sources of information. This is particularly relevant in pregnancy, where pain-related symptoms are common, but pharmacological treatment requires careful consideration of both maternal benefit and fetal safety (AlSaeed and Elmaghraby, 2021; Antonucci et al., 2012; Black et al., 2019).

The safety profile of analgesics during pregnancy differs according to the drug class, specific active substance, dose, indication, duration of exposure, and gestational timing. Non-steroidal anti-inflammatory drugs (NSAIDs), including ibuprofen, diclofenac, naproxen, and acetylsalicylic acid, have been associated with important fetal risks, particularly when used later in pregnancy. NSAID exposure in the second half of pregnancy has been linked to fetal renal dysfunction, oligohydramnios, and premature constriction or closure of the ductus arteriosus, while earlier exposure has been discussed in relation to spontaneous abortion and selected congenital outcomes (Antonucci et al., 2012; Black et al., 2019; Ericson and Källén, 2001; Bloor and Paech, 2013). However, NSAIDs should not be considered a pharmacologically or clinically uniform group in pregnancy. Acetylsalicylic acid is also classified as an NSAID, but low-dose aspirin has an established role in selected high-risk pregnancies for the prevention of pre-eclampsia and related placental complications when used according to clinical guidelines (Roberge et al., 2016). Therefore, counselling on analgesic safety in pregnancy should clearly distinguish between self-initiated analgesic use and medically indicated antiplatelet aspirin therapy.

Paracetamol is generally considered the preferred first-line analgesic during pregnancy when clinically indicated, particularly when used at the lowest effective dose and for the shortest possible duration (Bloor and Paech, 2013; Roberge et al., 2016; Toda, 2017; Scialli et al., 2010; Feldkamp et al., 2010). Nevertheless, it should not be presented as entirely risk-free or appropriate for unrestricted self-medication. Although the overall clinical experience with paracetamol in pregnancy is reassuring compared with many alternatives, rare case-series data have reported fetal ductus arteriosus constriction or closure following maternal paracetamol intake (Feldkamp et al., 2010). Such reports should be interpreted cautiously, especially because dose, duration, indication, concomitant exposures, and maternal clinical context may all influence risk assessment. They nevertheless reinforce the need for a balanced benefit–risk approach and appropriate professional counselling.

Beyond pharmacological safety itself, women’s knowledge and perceptions regarding analgesic use during pregnancy are highly relevant to clinical practice and public health. Previous studies have shown that pregnant women often identify paracetamol as the safest analgesic, but may have limited knowledge regarding NSAID classification, trimester-specific risks, and possible adverse fetal effects (Zaki and Albarraq, 2014; Raheel et al., 2017). The Saudi cross-sectional study on which the present work was methodologically modelled also found substantial uncertainty regarding the safety and side effects of analgesics used during pregnancy (AlSaeed and Elmaghraby, 2021). This is particularly relevant because the present study adapted that instrument to the Serbian context, including translation, contextual modification, and assessment of face and content validity after adaptation.

In Serbia, previous research has shown that medication use before and during pregnancy is common, indicating that drug exposure in this population represents an important pharmacoepidemiological and public health issue (Odalovic et al., 2012). However, more specific data on analgesic use patterns, self-medication, perceptions of safety, and knowledge gaps among women in Serbia remain limited. This evidence gap is important in the context of the widespread availability of non-prescription analgesics and the potential consequences of inappropriate use during pregnancy. Therefore, the aim of the present study was to assess patterns of analgesic use before and during pregnancy and to evaluate women’s knowledge and perceptions regarding the safety of analgesic use during pregnancy in Serbia.

## 2 Methods

### 2.1 Study design and setting

This study was designed as a cross-sectional survey aimed at assessing patterns of analgesic use before and during pregnancy, as well as women’s knowledge and perceptions regarding the safety of analgesic use during pregnancy in Serbia. The study targeted women living in the Republic of Serbia and was conducted using a self-administered structured questionnaire distributed electronically.

The methodological framework was based on a previously published cross-sectional study that assessed knowledge of analgesic drug utilization during pregnancy, with adaptation of the questionnaire and study content to the Serbian setting and to the aims of the present study.

### 2.2 Participants and eligibility criteria

Eligible participants were women aged 18 years or older residing in Serbia who were able to understand and complete the questionnaire in Serbian. Women were eligible if they were currently pregnant, including those in their first pregnancy, or if they had previous pregnancy experience. Thus, respondents with no previous pregnancies could still be eligible if they were pregnant at the time of survey completion.

Responses from women younger than 18 years, those not residing in Serbia, duplicate entries, and incomplete questionnaires were excluded from the final analysis.

### 2.3 Questionnaire development and adaptation

The questionnaire used in this study was adapted from a previously published instrument developed to assess women’s knowledge, perceptions, and use of analgesics during pregnancy (AlSaeed and Elmaghraby, 2021). The original questionnaire was translated into Serbian and contextually adapted for use in Serbia. The adaptation process included revision of wording, terminology, and the list of analgesic medicines to ensure linguistic clarity and relevance to the local clinical and regulatory context. The preliminary Serbian version was reviewed by the study investigators, who have expertise in pharmacology, toxicology, clinical pharmacology, and medication safety, in order to assess face and content validity. Before the main survey, the preliminary Serbian version of the questionnaire was pilot-tested among 10 women of reproductive age who met the general eligibility criteria for the target population. Participants were asked to comment on item clarity, comprehensibility, terminology, length, and technical functionality of the online form. No substantive changes to the conceptual structure or scoring system were made. Based on the feedback, minor wording changes were introduced to improve linguistic clarity and avoid ambiguity in selected items. Pilot responses were not included in the final analysis. The final questionnaire retained the conceptual structure of the source instrument and consisted of three sections: socio-demographic and reproductive characteristics, patterns of analgesic use before and during pregnancy, and a 19-item knowledge inventory regarding analgesic safety during pregnancy.

The final Serbian version of the questionnaire is available as Supplementary Material 1 to facilitate transparency and future use in comparable research settings.

### 2.4 Sample size

The sample size was estimated using an approach corresponding to the Raosoft® sample size calculator, with a margin of error of 5% and a confidence level of 95%. The population was set to the target subpopulation, namely women in the Republic of Serbia. According to estimates from the Statistical Office of the Republic of Serbia, the total number of women in Serbia is 3.382.745.

The sample size n and margin of error E were defined using the following relations:

$$x=Z^{2}\;r\left(100-r\right)$$

$$n=\frac{N\cdot x}{\left(N-1\right)E^{2}+x}E=\sqrt{\frac{\left(N-n\right)x}{n\left(N-1\right)}}$$

where N is the population size, r is the expected response distribution, assumed to be 50% to provide the most conservative estimate, and Z is the critical value for the confidence level c. For a 95% confidence level, Z = 1.96, while E = five (in percent).

First, the following value was calculated:

$$x=1.96^{2}\cdot 50\cdot \left(100-50\right)=3.8416\cdot 2500=9604$$

The sample size was then calculated as follows:

$$n=\frac{3,382,745\cdot 9604}{\left(3,382,745-1\right)\cdot 5^{2}+9604}=\frac{3,382,745\cdot 9604}{3,382,744\cdot 25+9604}n=\frac{32,489,882,980}{84,577,204}\approx 384.12$$

After rounding up, the minimum recommended sample size was 385 respondents. The final sample included 538 women, thus exceeding the minimum required sample size.

### 2.5 Recruitment and data collection procedure

The study was conducted from 15 March 2026 to 15 April 2026 using an anonymous, self-administered online questionnaire. A non-probability convenience sampling approach was used because participants were recruited through an electronically distributed survey link via social media platforms, including Facebook and Instagram. Eligible participants were women aged 18 years or older residing in Serbia who were currently pregnant or had previous pregnancy experience and were able to complete the questionnaire in Serbian. Before accessing the questionnaire, all potential participants were informed about the aims of the study, the voluntary nature of participation, and the confidentiality of data handling. No personally identifiable information was collected. Responses were screened before analysis, and duplicate entries, ineligible responses, and questionnaires with insufficiently complete data were excluded from the final dataset.

### 2.6 Outcome measures

The primary outcomes of the study were patterns of analgesic use before and during pregnancy, knowledge regarding the classification and safety of commonly used analgesics in pregnancy, perceived safety and effectiveness of specific analgesics, awareness of possible adverse effects, and the overall level of knowledge regarding analgesic use during pregnancy.

### 2.7 Knowledge score

The overall level of knowledge regarding analgesic use during pregnancy was assessed using a 19-item knowledge inventory. Each correct answer was assigned one point, while each incorrect answer was assigned 0 points. Total scores were calculated by summing the responses across all 19 items, resulting in an individual knowledge score for each respondent.

Consistent with the analytical approach used in the reference study, total knowledge scores were dichotomized into two categories: low level of knowledge and high level of knowledge. A score of less than 10 points was classified as low knowledge, whereas a score of 10 points or higher was classified as high knowledge. The proportions of respondents with low and high levels of knowledge were then determined. The cutoff of ≥10 correct answers was retained from the reference study to preserve methodological comparability (AlSaeed and Elmaghraby, 2021). This threshold approximately corresponds to achieving at least half of the possible score on the 19-item inventory. However, because this cutoff has not been independently validated in the Serbian population, this is now explicitly acknowledged as a limitation.

### 2.8 Statistical analysis

Data were coded and processed prior to analysis using Microsoft Excel. Statistical analyses were performed using *Jamovi* 2.5.4.0. Categorical variables were summarized using frequencies and percentages. To identify factors associated with a high level of knowledge regarding analgesic use during pregnancy, logistic regression analysis was performed. The dependent variable was the dichotomized knowledge level, classified as low (<10 points) or high (≥10 points). Independent variables included age, educational attainment, health-professional background, number of pregnancies, miscarriage experience, and use of analgesics before pregnancy. To avoid sparse data in regression analyses, selected variables were recategorized before modelling. Age was categorized as 35 years or younger versus 36 years or older; educational attainment as below college graduate versus college graduate and higher; number of pregnancies as fewer than 3 versus 3 or more; miscarriage experience as absent versus present; and use of analgesics before pregnancy as non-user versus user. Each variable was first assessed in a univariable logistic regression model. Crude odds ratios (ORs), 95% confidence intervals (CIs), and p-values were calculated. Variables with a crude p-value of less than 0.25 were entered into the multivariable logistic regression model, in line with the strategy used in the reference study. Adjusted odds ratios (aORs), 95% confidence intervals, and p-values were then reported. A two-sided p-value of less than 0.05 was considered statistically significant.

### 2.9 Ethical considerations

The study was conducted in accordance with the principles of the Declaration of Helsinki. This study was approved by the Ethics Committee of the Faculty of Medicine, University of Novi Sad (approval No. 01–39/33/1, dated 12 March 2026). Participation was voluntary and anonymous, and respondents were informed about the aims of the study and the confidentiality of data handling before accessing the questionnaire.

## 3 Results

### 3.1 Socio-demographic characteristics of respondents

A total of 538 women were included in the study. The largest age group comprised women aged 26–35 years (319/538, 59.3%), followed by those aged 36–45 years (109/538, 20.3%) and 19–25 years (80/538, 14.9%). Most respondents had completed bachelor-level education (315/538, 58.6%), while 204/538 (37.9%) had completed high school. Health professionals accounted for 21.6% of the sample (116/538). At the time of the survey, 160/538 respondents (29.7%) were pregnant. Regarding reproductive history, most women reported one pregnancy (203/538, 37.7%) or two pregnancies (181/538, 33.6%). Most respondents had no history of miscarriage (398/538, 74%), while 103/538 (19.1%) reported one miscarriage. Among women reporting miscarriage, 8/140 (5.7%) considered it drug-related, whereas 21/140 (15.0%) were unsure (Table 1).

**TABLE 1:** Socio-demographic profile of the respondents.

Socio-demographic characteristics	Count (n = 538)	Proportion, %
Age		
19–25 years	80	14.9
26–35 years	319	59.3
36–45 years	109	20.3
46 years and older	30	5.6
Educational attainment		
Elementary	4	0.7
High school	204	37.9
Bachelor	315	58.5
Master or doctorate	15	2.9
Health professional		
Yes	116	21.6
No	422	78.4
Present pregnancy status		
Yes	160	29.7
No	370	68.7
Do not know	8	1.5
Number of pregnancies		
Once	203	37.7
Twice	181	33.6
Thrice	76	14.1
Four or more times	31	5.8
No prior pregnancies	47	8.7
Number of miscarriages		
Never	398	74
Once	103	19.1
Twice	27	5
Three or more times	10	1.9
Drug-related miscarriage (n = 140)		
Yes	8	5.7
No	111	79.3
Do not know	21	15

To further contextualize the composition of the online convenience sample, selected respondent characteristics were descriptively compared with available Serbian population indicators. This comparison suggested that the age distribution of respondents was broadly comparable with national maternal age patterns, whereas women with higher educational attainment were overrepresented in the study sample (Supplementary Table S1).

### 3.2 Patterns of analgesic use before and during pregnancy

Overall, 278/538 respondents (51.7%) reported analgesic use during pregnancy. Among these women, 141/278 (50.7%) used analgesics during the first trimester. During pregnancy, analgesics were most commonly used following a physician’s recommendation (212/278, 76.3%), whereas 48/278 (17.3%) reported use on their own initiative. Paracetamol was by far the most frequently used analgesic during pregnancy (261/278, 93.9%), while the use of other analgesics was rare. Notably, 89/278 women (32.0%) reported using analgesics during pregnancy without prior consultation. Before pregnancy, analgesic use was reported by 447/538 respondents (83.1%). In this period, the most commonly used analgesics were paracetamol (231/447, 51.7%) and ibuprofen (175/447, 39.1%). Unlike use during pregnancy, pre-pregnancy analgesic use was most often initiated by the respondents themselves (205/447, 45.9%), followed closely by physician recommendation (186/447, 41.6%). Regarding awareness of adverse effects of analgesic use during pregnancy, 373/538 respondents (69.3%) stated that they had received some form of information (Table 2).

**TABLE 2:** Patterns of analgesic use before and during pregnancy and sources of safety-related information.

Variable	n (%)
Use of analgesics during pregnancy (N = 538)	
Yes	278 (51.7)
No	260 (48.3)
Use of analgesics during the first 3 months of pregnancy (n = 278)	
Yes	141 (50.7)
No	137 (49.3)
Recommendation/prescription during pregnancy (n = 278)	
Recommended by a physician	212 (76.3)
Recommended by a pharmacist	10 (3.6)
Recommended by another healthcare professional	4 (1.4)
Self-initiated	48 (17.3)
Recommended by family/friends	0 (0.0)
Other	4 (1.4)
Most commonly used analgesic during pregnancy (n = 278)	
Paracetamol	261 (93.9)
Ibuprofen	8 (2.9)
Diclofenac	2 (0.7)
Naproxen	0 (0.0)
Aspirin	4 (1.4)
Other	3 (1.1)
Without prior consultation (during pregnancy) (n = 278)	
Yes	89 (32.0)
No	189 (68.0)
Use of analgesics before pregnancy (N = 538)	
Yes	447 (83.1)
No	91 (16.9)
Most commonly used analgesic before pregnancy (n = 447)	
Paracetamol	231 (51.7)
Ibuprofen	175 (39.1)
Diclofenac	22 (4.9)
Naproxen	2 (0.4)
Aspirin	0 (0.0)
Other	17 (3.8)
Recommendation/prescription before pregnancy (n = 447)	
Recommended by a physician	186 (41.6)
Recommended by a pharmacist	41 (9.2)
Recommended by another healthcare professional	5 (1.1)
Self-initiated	205 (45.9)
Recommended by family/friends	8 (1.8)
Other	2 (0.4)
Awareness of adverse effects of analgesic use during pregnancy (n = 538)	
Yes	373 (69.3)
No	165 (30.7)
Source of information on adverse effects – first choice (n = 373)	
From a physician	70 (18.8)
From a pharmacist	40 (10.7)
From family/friends	13 (3.5)
Through social media	42 (11.3)
From professional literature	69 (18.5)
From another healthcare professional	7 (1.9)
Other	132 (35.4)

The category “Other” included responses such as the Embryotox website, patient information leaflets, Summary of Product Characteristics (SmPC), personal knowledge and experience, secondary medical school, pregnancy and breastfeeding education classes, ChatGPT, and gynecologist.

### 3.3 Knowledge of NSAID classification

**FIGURE 1:**
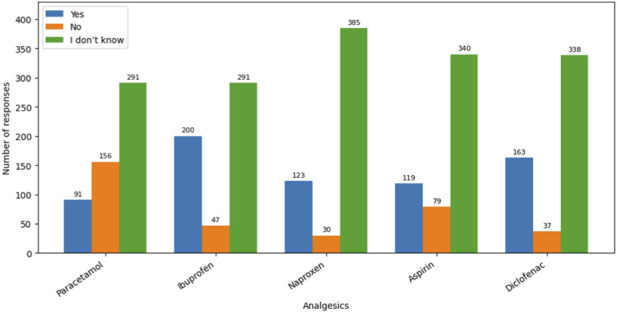
Classification of the following drugs as NSAIDs (non-steroidal anti-inflammatory drugs).

Considerable uncertainty was observed regarding the classification of commonly used analgesics as non-steroidal anti-inflammatory drugs (NSAIDs). For all listed drugs, the most frequent response was “I do not know”. This was particularly evident for naproxen (385/538, 71.6%), aspirin (340/538, 63.2%), and diclofenac (338/538, 62.8%). Among drugs that are NSAIDs, ibuprofen was most frequently correctly identified as such (200/538, 37.2%), followed by diclofenac (163/538, 30.3%). For paracetamol, which is not an NSAID, only 156/538 respondents (29.0%) correctly answered “no”, whereas 91/538 (16.9%) incorrectly identified it as an NSAID and 291/538 (54.1%) reported that they did not know (Figure 1).

### 3.4 Perceived safety and effectiveness of analgesics during pregnancy

After excluding “I do not know” responses, paracetamol was overwhelmingly perceived as both the safest and the most effective analgesic for use during pregnancy. It was identified as the safest analgesic by 482/496 respondents (97.2%) and as the most effective by 303/336 respondents (90.2%). All other drugs were selected only sporadically; for example, ibuprofen was chosen by 7/496 respondents (1.4%) as the safest and by 27/336 (8.0%) as the most effective analgesic during pregnancy (Table 3).

**TABLE 3:** Safety and effectiveness of analgesics during pregnancy.

Drugs	Safety (n/%)	Effectiveness (n/%)
Ibuprofen	7 (1.4)	27 (8.0)
Naproxen	0 (0.0)	1 (0.3)
Paracetamol	482 (97.2)	303 (90.2)
Aspirin	5 (1.0)	1 (0.3)
Diclofenac	2 (0.4)	4 (1.2)

### 3.5 Attitudes toward the risks of long-term analgesic use

**FIGURE 2:**
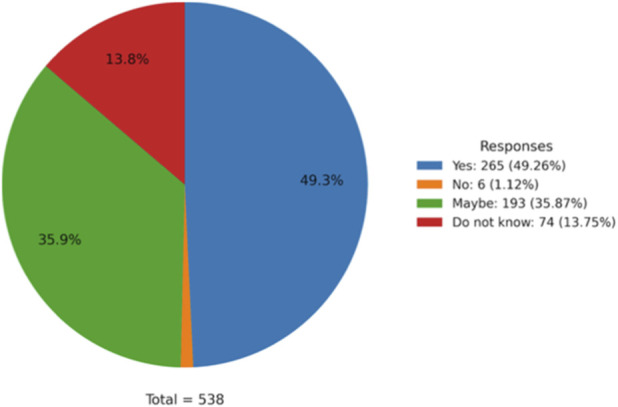
Long-term administration of the analgesic drug would negatively affect the fetal development.

When asked whether long-term use of analgesics could negatively affect fetal development, 265/538 respondents (49.3%) answered affirmatively. However, a substantial proportion remained uncertain, with 193/538 (35.9%) selecting “maybe” and 74/538 (13.8%) selecting “I do not know” (Figure 2).

**FIGURE 3:**
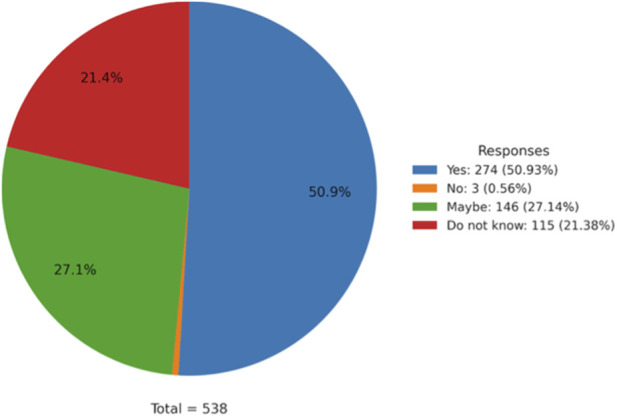
Long-term administration of analgesic drugs may cause stomach ulcers.

Similarly, regarding the potential of long-term analgesic use to cause stomach ulcers, 274/538 respondents (50.9%) believed that it could, whereas 146/538 (27.1%) answered “maybe” and 115/538 (21.4%) reported that they did not know (Figure 3).

### 3.6 Level of knowledge regarding analgesic use during pregnancy

Based on the predefined scoring system, most respondents had a low level of knowledge regarding analgesic use during pregnancy. Specifically, 350/538 women (65.1%) scored below 10 points, whereas 188/538 (34.9%) achieved a score of 10 or higher and were classified as having a high level of knowledge (Table 4).

**TABLE 4:** Distribution of respondents according to the level of knowledge regarding analgesics use during pregnancy.

Level of knowledge	Count (n = 538)	Relative frequency, %
Low (scores below 10 points)	350	65.1
High (scores of at least 10 points)	188	34.9

### 3.7 Factors associated with a high level of knowledge

In univariable logistic regression analysis, respondents aged 36 years or older had significantly higher odds of having a high level of knowledge compared with younger women (OR = 1.544, 95% CI 1.038–2.295, p = 0.032). However, this association was no longer statistically significant after adjustment (adjusted OR = 1.438, 95% CI 0.909–2.277, p = 0.121). The strongest association was observed for occupation in the health sector. Health professionals had markedly higher odds of having a high level of knowledge than non-health professionals in both univariable (OR = 12.196, 95% CI 7.417–20.053, p < 0.001) and multivariable analysis (adjusted OR = 12.468, 95% CI 7.502–20.724, p < 0.001). Educational attainment, number of pregnancies, miscarriage experience, and analgesic use before pregnancy were not independently associated with a high level of knowledge after adjustment. Although prior use of analgesics before pregnancy met the criterion for entry into the multivariable model, it was not retained as a significant predictor in the adjusted analysis (adjusted OR = 0.856, 95% CI 0.497–1.475, p = 0.576) (Table 5).

**TABLE 5:** Crude and adjusted odds ratios for factors associated with the level of knowledge regarding analgesic use during pregnancy.

Predictors	Crude OR	95% CI	p	Adjusted OR	95% CI	p
Age						
≤35 years	1	​	​	1	​	​
>35 years	1.544	1.038–2.295	0.032	1.438	0.909–2.277	0.121
Educational attainment						
Below college graduate	1	​	​	1	​	​
College graduate and higher	1.307	0.904–1.890	0.154	1.261	0.819–1.940	0.293
Medical-related profession						
Non-medical	1	​	​	1	​	​
Health worker	12.196	7.417–20.053	<0.001	12.468	7.502–20.724	<0.001
Number of pregnancies						
Less than 3	1	​	​	​	​	​
3 or more	1.200	0.775–1.859	0.414	​	​	​
Miscarriage experience						
Without	1	​	​	​	​	​
With	1.003	0.670–1.503	0.987	​	​	​
Use of analgesics before pregnancy						
Non-user	1	​	​	1	​	​
User	1.421	0.867–2.329	0.163	0.856	0.497–1.475	0.576

In the adjusted model, only predictors with a crude p-value <0.25 were included.

## 4 Discussion

This study adds a more focused view of analgesic use in pregnancy in Serbia by examining not only patterns of use, but also women’s understanding of medication safety. Several findings stand out. Analgesic use during pregnancy was common, including use in the first trimester, and paracetamol clearly predominated as the analgesic most often used and most often perceived as both the safest and the most effective option. At the same time, almost one-third of women who used analgesics during pregnancy reported doing so without prior consultation, and most respondents had a low overall level of knowledge regarding analgesic safety.

The overall frequency of analgesic use observed in our sample is in line with the broader literature showing that medication exposure during pregnancy is common and that analgesics belong among the medications most often used by pregnant women (Antonucci et al., 2012; Black et al., 2019; Odalovic et al., 2012; Lupattelli et al., 2014). In Serbia, previous studies conducted over a decade ago have already shown that drug use before and during pregnancy is frequent, with differences between the pre-pregnancy and pregnancy periods reflecting both changing clinical needs and changing prescribing patterns (Odalovic et al., 2012; Odalovic et al., 2013). Similar findings have been reported in multinational studies, although direct comparison is not straightforward because estimates vary depending on study design, setting, recall period, and whether prescription and non-prescription products are analysed together (Lupattelli et al., 2014). Still, our data suggest that analgesic exposure during pregnancy is far from rare in Serbia and should be considered an important component of routine medication counseling in antenatal care.

The dominant position of paracetamol in our results is not surprising. It is consistent with earlier studies showing that paracetamol is usually the analgesic most commonly taken during pregnancy and the one most often regarded as acceptable when an analgesic is needed (Antonucci et al., 2012; Black et al., 2019; Bloor and Paech, 2013; Toda, 2017; Scialli et al., 2010; Kassaw and Wabe, 2012; Zafeiri et al., 2021). In our sample, this pattern was especially clear, both in actual use and in safety perception. That is reassuring to a degree, since paracetamol is generally considered the preferred first-line analgesic during pregnancy when clinically indicated and used at the lowest effective dose for the shortest appropriate time (Bloor and Paech, 2013; Toda, 2017; Scialli et al., 2010). At the same time, this result should not be interpreted too optimistically. A correct preference for paracetamol does not necessarily mean that women understand the broader safety profile of analgesics in pregnancy. In our study, that gap was evident in the marked uncertainty surrounding NSAID classification and risk.

This gap is clinically important. Many respondents were unsure whether commonly used drugs such as ibuprofen, diclofenac, naproxen, and aspirin belong to the NSAID group, and “I do not know” was often the dominant answer. That finding echoes earlier reports showing that pregnant women frequently recognize paracetamol as the safest option, but have a much less secure understanding of NSAIDs, their classification, and the periods of pregnancy in which they may be problematic (Zaki and Albarraq, 2014; Raheel et al., 2017; Kassaw and Wabe, 2012; Damase-Michel et al., 2009; Undela et al., 2021). This matters because the risk profile of analgesics is not uniform across drug classes or across gestation. Earlier exposure to NSAIDs has been discussed in relation to spontaneous abortion, whereas later exposure has been associated with premature closure of the ductus arteriosus, oligohydramnios, neonatal renal impairment, and other adverse fetal outcomes (Antonucci et al., 2012; Black et al., 2019; Ericson and Källén, 2001; Bloor and Paech, 2013). In practice, women do not need a detailed pharmacology lecture, but they do need to understand one basic point: not all common pain relievers are interchangeable during pregnancy, even if they are easy to obtain.

Another finding that deserves attention is the proportion of women who used analgesics during pregnancy without prior consultation. Although the rate in our study was lower than in some published reports, it remains substantial. International data show considerable variation in self-medication and unsupervised medication use during pregnancy. For example, the recent Saudi analgesic-focused study reported a considerably higher proportion of women using analgesics without referral to a physician or pharmacist (AlSaeed and Elmaghraby, 2021). Other studies have described lower rates in Indonesia and higher rates in Tanzania, Pakistan, and Croatia (Ahmed et al., 2020; Atmadani et al., 2020; Marwa et al., 2018; Bohio et al., 2016; Jovanović and Vulić, 2025). Such variation is not unexpected. It probably reflects differences in healthcare access, local prescribing culture, OTC availability, health literacy, and the kinds of symptoms women choose to manage on their own. Even so, the larger message is consistent: self-directed use of medications in pregnancy is common enough to require active attention from healthcare professionals rather than being treated as a marginal issue.

The very high prevalence of analgesic use before pregnancy in our sample is also relevant when interpreting these findings. Previous Serbian research showed that medication use before pregnancy is one of the strongest predictors of medication use during pregnancy (Odalovic et al., 2013). Our results fit well with that observation despite the fact that previous studies were conducted more than a decade ago. Analgesic use before pregnancy was very common, and before pregnancy it was often self-initiated. This suggests that women may enter pregnancy with already established habits of using OTC pain medication for everyday symptoms such as headache, fever, or dysmenorrhea. Those habits do not automatically disappear once pregnancy begins. For that reason, preconception counseling and very early pregnancy counseling may be just as important as later antenatal advice. If medication habits are addressed only after pregnancy is well established, part of the opportunity for prevention may already have been missed.

One of the more revealing aspects of our study was the mismatch between reported access to information and measured knowledge. Although many women stated that they had received some information about the adverse effects of analgesic use during pregnancy, two-thirds still fell into the low-knowledge category. This suggests that the central problem is not simply whether information is available, but whether it is clear, clinically meaningful, and obtained from reliable sources. This interpretation is supported by the distribution of information sources in our sample, where physicians accounted for only a minority of first-choice sources and “other” sources were most frequently selected. Previous studies have shown that pregnant women often seek medication information online and from multiple informal channels (Sinclair et al., 2018; De Santis et al., 2010; Hämeen-Anttila et al., 2015; Grimes et al., 2014; Bjelke et al., 2016). Sinclair et al. found that women frequently used the internet to search for medication safety information during pregnancy and that analgesics were among the most commonly searched categories (Sinclair et al., 2018). Other studies have similarly shown that pregnant women use the internet to fill perceived gaps in professional counseling, although the quality of available information is variable (De Santis et al., 2010; Grimes et al., 2014; Bjelke et al., 2016). This makes fragmented understanding almost inevitable: women may remember a general message, but not the details that make it clinically useful.

The role of pharmacists deserves particular attention here. In theory, pharmacists are in an ideal position to provide timely advice about OTC analgesics, especially because they are often the first health professionals encountered when a woman wants immediate symptom relief. In practice, however, this role is not always fully developed. A comparative Serbian–Norwegian study showed that pharmacists’ counseling of pregnant women with common ailments varied considerably and that advice was not always optimal or consistent (Odalović et al., 2016). That is important for interpreting our own data, where pharmacists were only a modestly represented information source. Better use of pharmacists in pregnancy-related medication counseling could be valuable, but it would likely require more structured education and clearer practice-oriented guidance. This may be especially important in settings where women frequently rely on non-prescription medicines and informal advice.

The regression analysis in our study offers one additional point that is worth stressing. Health-professional background was the strongest predictor of better knowledge, whereas age and education lost statistical significance after adjustment. This suggests that general education alone does not guarantee medication literacy in pregnancy. Women may be highly educated and still feel uncertain about what is safe, what should be avoided, and when professional advice is needed. That interpretation is consistent with earlier work showing that pregnant women often overestimate medication risks or feel uncertain even when dealing with relatively common medicines (Undela et al., 2021; Nordeng et al., 2010; Bonari et al., 2005; Ceulemans et al., 2019). From a clinical perspective, this means that counseling should not assume prior understanding. It should be simple, explicit, and practical, regardless of the woman’s formal educational level.

Taken together, our findings suggest that the main problem is not a complete lack of awareness, but a pattern of partial knowledge. Women in this sample generally recognized paracetamol as the preferred analgesic in pregnancy, which is encouraging. However, this was accompanied by substantial uncertainty regarding NSAIDs, frequent reliance on self-initiated pre-pregnancy use patterns, and a notable proportion of analgesic use during pregnancy without prior consultation. In that sense, the issue is less about isolated misinformation and more about incomplete medication literacy. Future efforts should therefore move beyond general warnings and focus on a few concrete messages: that not all analgesics are equivalent in pregnancy, that NSAID-related risks depend on timing, and that even familiar OTC medications should ideally be discussed with a healthcare professional once pregnancy is suspected or confirmed. Better preconception counseling, clearer antenatal education, more visible pharmacist involvement, and easier access to trustworthy information sources may all help reduce avoidable uncertainty and improve medication safety in pregnancy in Serbia (Odalovic et al., 2013; Sinclair et al., 2018; Hämeen-Anttila et al., 2015; Odalović et al., 2016; de Jonge et al., 2015).

## 5 Limitations

This study has several limitations. First, its cross-sectional design does not allow causal inferences. Second, the data were based on self-report collected through an electronic questionnaire, which introduces the possibility of recall and reporting bias, particularly for previous pregnancies, miscarriage history, and analgesic use before or during pregnancy. Third, the use of an online survey and convenience sampling may have limited the representativeness of the sample. Women without regular internet access, lower digital literacy, or lower engagement with online platforms may have been underrepresented, which may reduce the generalizability of the findings to the broader population of women in Serbia. In addition, because the questionnaire was distributed electronically, a response rate could not be reliably determined. Finally, although the questionnaire was adapted from a previously published instrument, it was not newly developed and fully psychometrically validated specifically for the Serbian population. The dichotomization of the 19-item knowledge score may also have simplified a more nuanced spectrum of knowledge. Despite these limitations, the study provides useful insight into analgesic use patterns, safety perceptions, and knowledge gaps among women in Serbia, and may serve as a basis for future research using broader and more representative sampling strategies. Because participants were recruited online using a non-probability convenience sampling approach, the sample should not be considered nationally representative of all pregnant women or women with pregnancy experience in Serbia. Women with higher digital literacy, greater interest in pregnancy-related health topics, or better access to social media may have been overrepresented.

## 6 Conclusion

In this online convenience sample of women in Serbia, analgesic use during pregnancy was common, and a substantial proportion of respondents reported use without prior consultation. Although paracetamol was widely recognized as the preferred analgesic during pregnancy, knowledge regarding analgesic safety, particularly NSAID-related risks, was limited. These findings should be interpreted in light of the non-probability sampling design and support the need for clearer counselling and targeted educational interventions.

## Data Availability

The raw data supporting the conclusions of this article will be made available by the authors, without undue reservation.
