# Supplementary material for: Use of analgesics during pregnancy: patterns of use and knowledge of medication safety among women in Serbia
Source: Front Pharmacol. 2026 Jun 18;17:1874369. doi: 10.3389/fphar.2026.1874369 (PMC13323017; doi:10.3389/fphar.2026.1874369)
Supplement: Supplementary file 1 [file Supplementaryfile1.docx]

**Supplementary File 1. English version of the questionnaire**

**Use of analgesics during pregnancy: assessment of patterns of use and level of knowledge regarding the safety of their use**

**I. Socio-demographic and reproductive data**

1. **How old are you?**
   ☐ 18–25
   ☐ 26–35
   ☐ 36–45
   ☐ 46 years or older
2. **What is your highest level of education?**
   ☐ Elementary school
   ☐ High school
   ☐ University degree
   ☐ Doctorate
3. **Are you a healthcare professional?**
   ☐ Yes
   ☐ No
4. **Current pregnancy status: Are you currently pregnant?**
   ☐ Yes
   ☐ No
   ☐ I do not know
5. **Number of pregnancies you have had:**
   ☐ None
   ☐ 1
   ☐ 2
   ☐ 3
   ☐ 4 or more
6. **Have you had a miscarriage?**
   ☐ No
   ☐ 1
   ☐ 2
   ☐ 3 or more
7. **Was the miscarriage related to medication use?**
   ☐ Yes
   ☐ No
   ☐ I do not know

**II. Use of analgesics before pregnancy**

1. **Did you use analgesics before pregnancy?**
   ☐ Yes
   ☐ No
2. **On whose recommendation did you use analgesics before pregnancy?**
   Multiple answers may be selected.
   ☐ On a physician’s recommendation
   ☐ On a pharmacist’s recommendation
   ☐ On the recommendation of another healthcare professional
   ☐ On my own initiative
   ☐ On the recommendation of family/friends
   ☐ Other: __________
3. **For which conditions did you most commonly use analgesics before pregnancy?**
   Multiple answers may be selected.
   ☐ Headache
   ☐ Toothache
   ☐ Fever
   ☐ Rheumatic pain
   ☐ Menstrual pain
   ☐ Other: __________
4. **Which analgesics did you most commonly use before pregnancy?**
   Multiple answers may be selected.
   ☐ Paracetamol/acetaminophen (Panadol Advance)
   ☐ Ibuprofen (Zentidol, Brufen, Rapidol S, Rozyfen, Caffetin menstrual)
   ☐ Diclofenac (Rapten Duo, Diklofen, Rapten-K)
   ☐ Naproxen (Naproksen HF, Nalgesin S, Napex)
   ☐ Acetylsalicylic acid (Aspirin)
   ☐ Other: __________

**III. Use of analgesics during pregnancy**

1. **Did you use analgesics during pregnancy?**
   ☐ Yes
   ☐ No
2. **Did you use analgesics during the first 3 months of pregnancy?**
   ☐ Yes
   ☐ No
3. **On whose recommendation did you use analgesics during pregnancy?**
   Multiple answers may be selected.
   ☐ On a physician’s recommendation
   ☐ On a pharmacist’s recommendation
   ☐ On the recommendation of another healthcare professional
   ☐ On my own initiative
   ☐ On the recommendation of family/friends
   ☐ Other: __________
4. **Which analgesic did you most commonly use during pregnancy?**
   Multiple answers may be selected.
   ☐ Paracetamol/acetaminophen (Panadol Advance)
   ☐ Ibuprofen (Zentidol, Brufen, Rapidol S, Rozyfen, Caffetin menstrual)
   ☐ Diclofenac (Rapten Duo, Diklofen, Rapten-K)
   ☐ Naproxen (Naproksen HF, Nalgesin S, Napex)
   ☐ Acetylsalicylic acid (Aspirin)
   ☐ Other: __________
5. **For which conditions did you most commonly use analgesics during pregnancy?**
   Multiple answers may be selected.
   ☐ Headache
   ☐ Toothache
   ☐ Fever
   ☐ Rheumatic pain
   ☐ Other: __________
6. **Did you use analgesics during pregnancy without prior consultation with a physician or pharmacist?**
   ☐ Yes
   ☐ No
7. **Were you informed about the possible adverse effects of analgesics during pregnancy?**
   ☐ Yes
   ☐ No
8. **Where did you obtain information about the possible adverse effects of analgesics during pregnancy?**
   Multiple answers may be selected.
   ☐ From a physician
   ☐ From a pharmacist
   ☐ From another healthcare professional
   ☐ From family/friends
   ☐ Through social media
   ☐ From scientific/professional literature
   ☐ Other: __________

**IV. Knowledge and attitudes regarding the safety of analgesics during pregnancy**

1. **Do analgesics have anti-inflammatory properties?**
   ☐ Yes
   ☐ No
   ☐ I do not know
2. **Does paracetamol/acetaminophen (Panadol Advance) belong to NSAIDs, i.e. non-steroidal anti-inflammatory drugs?**
   ☐ Yes
   ☐ No
   ☐ I do not know
3. **Does ibuprofen (Zentidol, Brufen, Rapidol S, Rozyfen, Caffetin menstrual) belong to NSAIDs, i.e. non-steroidal anti-inflammatory drugs?**
   ☐ Yes
   ☐ No
   ☐ I do not know
4. **Does diclofenac (Rapten Duo, Diklofen, Rapten-K) belong to NSAIDs, i.e. non-steroidal anti-inflammatory drugs?**
   ☐ Yes
   ☐ No
   ☐ I do not know
5. **Does naproxen (Naproksen HF, Nalgesin S, Napex) belong to NSAIDs, i.e. non-steroidal anti-inflammatory drugs?**
   ☐ Yes
   ☐ No
   ☐ I do not know
6. **Does acetylsalicylic acid (Aspirin) belong to NSAIDs, i.e. non-steroidal anti-inflammatory drugs?**
   ☐ Yes
   ☐ No
   ☐ I do not know
7. **Which analgesic is the safest for use during pregnancy?**
   ☐ Paracetamol/acetaminophen (Panadol Advance)
   ☐ Ibuprofen (Zentidol, Brufen, Rapidol S, Rozyfen, Caffetin menstrual)
   ☐ Diclofenac (Rapten Duo, Diklofen, Rapten-K)
   ☐ Naproxen (Naproksen HF, Nalgesin S, Napex)
   ☐ Acetylsalicylic acid (Aspirin)
   ☐ I do not know
8. **Which analgesic is the most effective for pain relief during pregnancy?**
   ☐ Paracetamol/acetaminophen (Panadol Advance)
   ☐ Ibuprofen (Zentidol, Brufen, Rapidol S, Rozyfen, Caffetin menstrual)
   ☐ Diclofenac (Rapten Duo, Diklofen, Rapten-K)
   ☐ Naproxen (Naproksen HF, Nalgesin S, Napex)
   ☐ Acetylsalicylic acid (Aspirin)
   ☐ I do not know
9. **Can the use of analgesics during the last 3 months of pregnancy have harmful effects?**
   ☐ Yes
   ☐ No
   ☐ Maybe
   ☐ I do not know
10. **Which analgesic is the safest for use during the last 3 months of pregnancy?**
    ☐ Paracetamol/acetaminophen (Panadol Advance)
    ☐ Ibuprofen (Zentidol, Brufen, Rapidol S, Rozyfen, Caffetin menstrual)
    ☐ Diclofenac (Rapten Duo, Diklofen, Rapten-K)
    ☐ Naproxen (Naproksen HF, Nalgesin S, Napex)
    ☐ Acetylsalicylic acid (Aspirin)
    ☐ I do not know
11. **Can long-term use of analgesics negatively affect fetal development?**
    ☐ Yes
    ☐ No
    ☐ Maybe
    ☐ I do not know
12. **Can long-term use of analgesics cause gastric ulcer?**
    ☐ Yes
    ☐ No
    ☐ Maybe
    ☐ I do not know
13. **Which of the listed medicines can cause gastric ulcer?**
    Multiple answers may be selected.
    ☐ Paracetamol/acetaminophen (Panadol Advance)
    ☐ Ibuprofen (Zentidol, Brufen, Rapidol S, Rozyfen, Caffetin menstrual)
    ☐ Diclofenac (Rapten Duo, Diklofen, Rapten-K)
    ☐ Naproxen (Naproksen HF, Nalgesin S, Napex)
    ☐ Acetylsalicylic acid (Aspirin)
    ☐ I do not know
14. **Can paracetamol/acetaminophen cause allergic reactions?**
    ☐ Yes
    ☐ No
    ☐ I do not know

**Note:** This supplementary file presents the English translation of the Serbian questionnaire used in the study. The questionnaire included socio-demographic and reproductive characteristics, patterns of analgesic use before and during pregnancy, sources of information, and items assessing knowledge regarding analgesic classification and safety during pregnancy. Multiple-response questions are indicated where applicable.
