# Supplementary material for: Use of analgesics during pregnancy: patterns of use and knowledge of medication safety among women in Serbia
Source: Front Pharmacol. 2026 Jun 18;17:1874369. doi: 10.3389/fphar.2026.1874369 (PMC13323017; doi:10.3389/fphar.2026.1874369)
Supplement: Supplementary file 2 [file Table1.docx]

Supplementary Table S1. Descriptive comparison of the study sample with available Serbian population indicators

| **Characteristic** | **Study sample, n/N (%)** | **Serbian reference indicator** | **Direction of difference** |
| --- | --- | --- | --- |
| **Age group** |  |  |  |
| ≤25 years | 80/538 (14.9) | Approx. 18.6% of live births in Serbia occurred among mothers aged ≤24 years in 2023 | Slight underrepresentation of younger women |
| 26–35 years | 319/538 (59.3) | Approx. 59.0% of live births occurred among mothers aged 25–34 years in 2023 | Broadly comparable, although age categories are not identical |
| >35 years | 139/538 (25.8) | Approx. 22.4% of live births occurred among mothers aged ≥35 years in 2023 | Slight overrepresentation of older women |
| **Educational attainment** |  |  |  |
| Elementary or lower | 4/538 (0.7) | Among women aged ≥15 years in Serbia, 27.4% had elementary education or less in the 2022 Census | Marked underrepresentation of women with lower education |
| High school | 204/538 (37.9) | Among women aged ≥15 years in Serbia, 48.1% had completed secondary education in the 2022 Census | Underrepresentation of women with secondary education |
| Bachelor, master, or doctorate | 330/538 (61.3) | Among women aged ≥15 years in Serbia, 24.0% had higher education in the 2022 Census | Marked overrepresentation of highly educated women |
| **Health-professional background** | 116/538 (21.6) | No directly comparable national benchmark was identified | Likely overrepresentation of health-literate respondents |
| **Currently pregnant at survey completion** | 160/538 (29.7) | No directly comparable benchmark for an online sample of pregnant and previously pregnant women | Not directly assessable |
| **Pregnancy history** |  |  |  |
| One pregnancy | 203/538 (37.7) | No directly comparable national benchmark because the study measured number of pregnancies, not live-birth order | Not directly comparable |
| Two pregnancies | 181/538 (33.6) | Not directly comparable | Not directly comparable |
| Three or more pregnancies | 107/538 (19.9) | Not directly comparable | Not directly comparable |
| No prior pregnancies, but currently pregnant | 47/538 (8.7) | Not directly comparable | Not directly comparable |
| **Miscarriage history** | 140/538 (26.0) | No directly comparable national benchmark was identified | Not directly assessable |

**Note**: Percentages in the study sample are based on the 538 respondents included in the final analysis. Serbian age-reference indicators were calculated descriptively from the distribution of live births by maternal age in the Statistical Office of the Republic of Serbia data for 2023. Exact comparability is limited because the study used age categories of 19–25, 26–35, 36–45, and ≥46 years, whereas national birth statistics are reported in standard five-year maternal age groups. Educational benchmarks are based on the 2022 Census distribution of educational attainment among women aged ≥15 years and should be interpreted as an indirect comparison, because they refer to the general female population rather than specifically to pregnant women or women with pregnancy experience. National statistics reported 61,052 live births in Serbia in 2023 and provide live-birth counts by maternal age; census data show that 24.03% of women aged ≥15 years had high or higher education, while 48.14% had secondary education and 27.44% had elementary education or less.
